# Albuminuria

**Published:** 1883-08

**Authors:** 


### Albuminuria.

The dependence of the symptom albuminuria on the existence of a dyscrasia of the whole system is a view which cannot be said to be of interest for its novelty. M. Semmola recently advocated, at a meeting of the Academy of Medicine, the ancient doctrine that Bright's disease was a malady of the whole body, that the albuminuria was the result of a cachexia, and preceded the renal changes. Various forms of albumen were injected into the vessels of animals with the production of albuminuria, and it was found that the kidneys exhibited anatomical alterations similar to those met with in the various phases of Bright's disease. As the writer in the *Gazette Medicale* suggests, it is probable that the appearance in the renal circulation of albumens alien to those of the animal in question may, like any other irritant, induce morbid changes of an inflammatory nature. Even were the morbid appearances of the kidneys precisely the same in the experimental and clinical disease, still no argument of any weight would be afforded, seeing that many different causes may produce like effects—*The London Lancet.*

